# Disruption of the mouse *Shmt2* gene confers embryonic anaemia via foetal liver-specific metabolomic disorders

**DOI:** 10.1038/s41598-019-52372-6

**Published:** 2019-11-05

**Authors:** Haruna Tani, Takayuki Mito, Vidya Velagapudi, Kaori Ishikawa, Moe Umehara, Kazuto Nakada, Anu Suomalainen, Jun-Ichi Hayashi

**Affiliations:** 1 Graduate School of Life and Environmental Sciences, University of Tsukuba, 1-1-1 Tennodai, Tsukuba, Ibaraki 305-8572 Japan; 2 Japan Society for the Promotion of Science (JSPS), 8 Ichiban-cho, Chiyoda-ku, Tokyo 102-8472 Japan; 3 Stem Cells and Metabolism Research Program, Faculty of Medicine, University of Helsinki, 00290 Helsinki, Finland; 4 Metabolomics Unit, Institute for Molecular Medicine Finland FIMM, HiLIFE, University of Helsinki, 00290 Helsinki, Finland; 5 Faculty of Life and Environmental Sciences, University of Tsukuba, 1-1-1 Tennodai, Tsukuba, Ibaraki 305-8572 Japan; 6 Neuroscience Center, University of Helsinki, 00290 Helsinki, Finland; 7 Life Science Center for Survival Dynamics, Tsukuba Advanced Research Alliance (TARA), University of Tsukuba, 1-1-1 Tennodai, Tsukuba, Ibaraki 305-8577 Japan

**Keywords:** Energy metabolism, Mitochondria

## Abstract

In a previous study, we proposed that age-related mitochondrial respiration defects observed in elderly subjects are partially due to age-associated downregulation of nuclear-encoded genes, including *serine hydroxymethyltransferase 2* (*SHMT2*), which is involved in mitochondrial one-carbon (1C) metabolism. This assertion is supported by evidence that the disruption of mouse *Shmt2* induces mitochondrial respiration defects in mouse embryonic fibroblasts generated from *Shmt*2-knockout E13.5 embryos experiencing anaemia and lethality. Here, we elucidated the potential mechanisms by which the disruption of this gene induces mitochondrial respiration defects and embryonic anaemia using *Shmt2*-knockout E13.5 embryos. The livers but not the brains of *Shmt2*-knockout E13.5 embryos presented mitochondrial respiration defects and growth retardation. Metabolomic profiling revealed that *Shmt2* deficiency induced foetal liver-specific downregulation of 1C-metabolic pathways that create taurine and nucleotides required for mitochondrial respiratory function and cell division, respectively, resulting in the manifestation of mitochondrial respiration defects and growth retardation. Given that foetal livers function to produce erythroblasts in mouse embryos, growth retardation in foetal livers directly induced depletion of erythroblasts. By contrast, mitochondrial respiration defects in foetal livers also induced depletion of erythroblasts as a consequence of the inhibition of erythroblast differentiation, resulting in the manifestation of anaemia in *Shmt*2-knockout E13.5 embryos.

## Introduction

Human aging and age-related mitochondrial respiration defects are thought to be caused by the age-associated accumulation of somatic mutations in mitochondrial DNA (mtDNA)^1–7^. In contrast to this postulate, our previous studies^8,9^ proposed a different concept of human aging: age-related mitochondrial respiration defects observed in the fibroblasts of elderly subjects^10^ are not due to the accumulation of somatic mutations in mtDNA but to epigenetic downregulation of nuclear-encoded genes, including *serine hydroxymethyltransferase 2* (*SHMT2*), involved in mitochondrial one-carbon (1C) metabolism^11^. Indeed, remodelled 1C metabolism was recently linked to mitochondrial respiration defects and mitochondrial diseases^12,13^.

The concept that mitochondrial respiration defects in elderly subjects are due partly to epigenetic downregulation of nuclear-encoded *SHMT2*^8^ is supported by our subsequent findings that knockout of mouse *Shmt2* induces mitochondrial respiration defects in mouse embryonic fibroblasts (MEFs) obtained from *Shmt2*-knockout embryos and confers embryonic lethality with anaemia^14^. Moreover, recent reports^15–17^ revealed that knockout of human *SHMT2* in cultivated human cell lines induces respiration defects as a result of deprivation of the products of mitochondrial 1C metabolism, including taurinomethyluridine bases^15^ and formylmethionyl-tRNA (f-Met tRNA)^16^, required to translate mtDNA-encoded subunits of mitochondrial respiratory chain complexes. Therefore, the occurrence of age-related mitochondrial respiration defects in elderly subjects might be partially explained by the age-associated downregulation of *SHMT2*^8^ and the resulting downregulation of mitochondrial 1C metabolism.

The knockout of other genes involved in mitochondrial 1C metabolism sometimes induces embryonic lethality in mice with either anaemia^18^ or neural tube defects (NTDs)^19–22^, indicating induction of tissue-specific onset of the disorders in mouse embryos by the disruption of these genes. In the case of *Shmt2*-knockout embryos, macroscopic abnormalities include anaemia and small embryonic sizes^14^. Given that foetal livers function as sites for erythropoiesis (differentiation of erythroblasts) in mouse embryos^23^, the presence of respiration defects in the foetal livers of *Shmt2*-knockout embryos would be related to the manifestation of anaemia. Moreover, considering that not only mitochondrial respiration defects but also growth retardation are commonly observed in *Shmt2*-knockout MEFs^14^ and in *SHMT2*-knockout human cell lines^15–17^, growth retardation alongside mitochondrial respiration defects in the foetal livers of *Shmt2*-knockout embryos would also be involved in the manifestation of anaemia.

Here, we studied the livers and brains of *Shmt2*-knockout E13.5 embryos to identify the potential mechanisms by which *Shmt2* disruption induces mitochondrial respiration defects and growth retardation preferentially in foetal livers and confers anaemia in *Shmt2*-knockout E13.5 embryos.

## Results

### Respiration defects and growth retardation in *Shmt2*-knockout foetal livers

We previously identified that MEFs obtained from *Shmt2*-knockout E13.5 mouse embryos present mitochondrial respiration defects^14^. Moreover, they manifest significant growth retardation in comparison to MEFs obtained from wildtype E13.5 embryos^14^. In this study, we examined whether foetal tissues—specifically, livers and brains from *Shmt2*-knockout E13.5 embryos—also exhibit these abnormalities.

**Figure 1:**
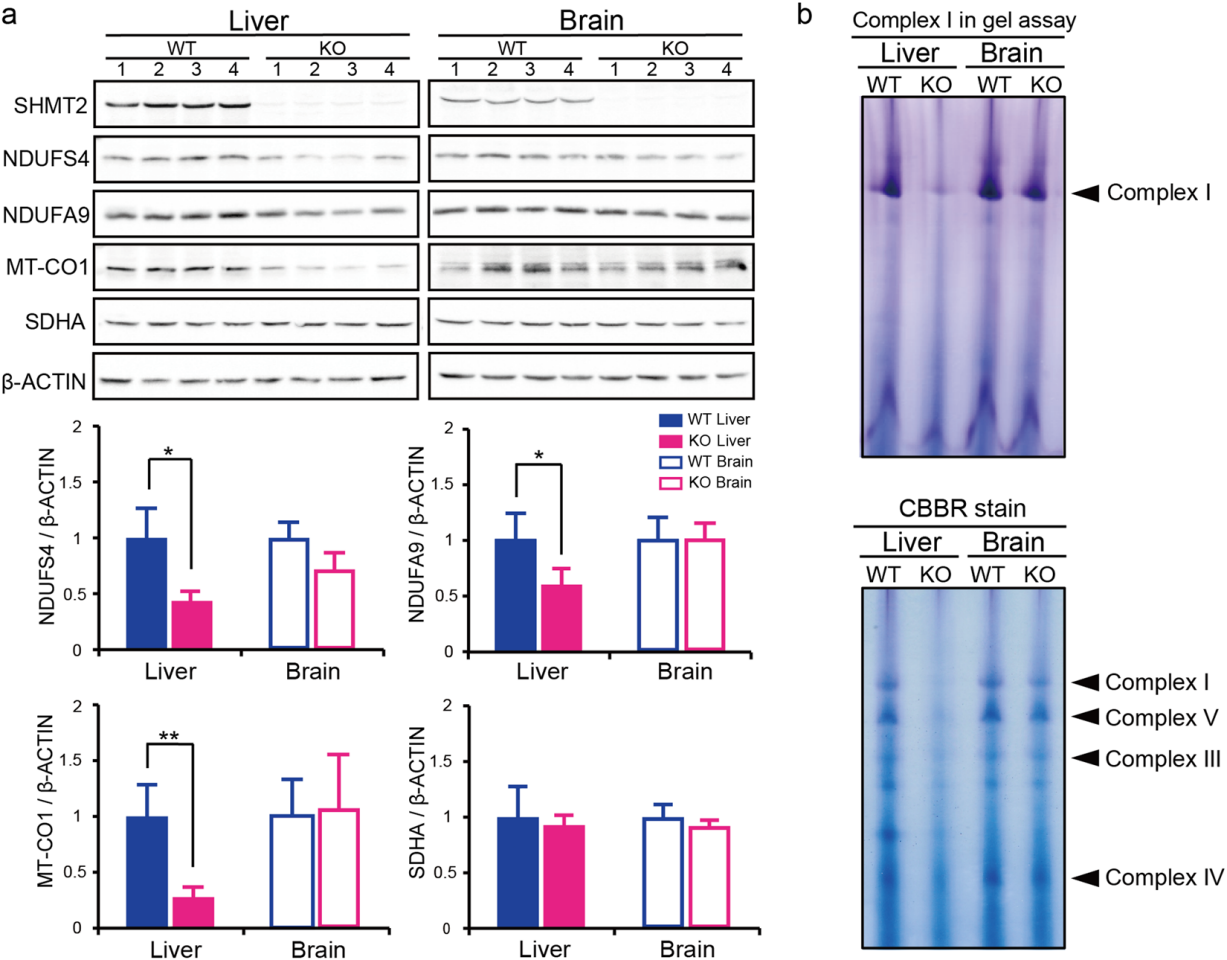
Effects of *Shmt2* knockout on mitochondrial respiratory chain complexes of livers and brains from E13.5 embryos. (**a**) Western blot analysis to estimate the quantity of the subunits comprising the mitochondrial respiratory chain complexes for comparison between wild-type and *Shmt2*-knockout foetal tissues. SHMT2, SHMT2 protein encoded by *Shmt2*; NDUSF4 and NDUFA9, nuclear DNA-encoded subunits of respiratory chain complex I; MT-CO1, an mtDNA-encoded subunit of respiratory chain complex IV; SDHA, a nuclear DNA-encoded subunit of respiratory chain complex II. Filled blue bars, wild-type foetal livers; filled red bars, *Shmt2*-knockout foetal livers; open blue bars, wild-type foetal brains; open red bars, *Shmt2*-knockout foetal brains. Data represent the mean ± S.D. *P < 0.05; **P < 0.01, Student’s *t* test (wild-type tissues, n = 4; tissues without *Shmt2*, n = 4). (**b**) In-gel evaluation of the activity of mitochondrial respiratory chain complex I, and detection of mitochondrial respiratory chain complexes I, III, IV, and V by blue native PAGE. To detect respiratory chain complexes, gels were stained with Coomassie Brilliant Blue R250 (CBBR).

Western blot analysis of livers from *Shmt2*-knockout E13.5 embryos (Figs 1a and S1) showed decreased levels of not only an mtDNA-encoded subunit (MT-CO1) but also some nuclear DNA-encoded subunits (NDUFS4 and NDUFA9) comprising mitochondrial respiratory chain complexes. By contrast, foetal brains did not show these abnormalities (Fig. 1a). Moreover, in-gel activity assays showed preferential reduction in the activity of mitochondrial respiratory chain complex I in *Shmt2*-knockout livers but not in brains (Fig. 1b). Additionally, blue native gel analysis showed preferential reductions in the amounts of mitochondrial respiratory chain complexes I, III, IV, and V in *Shmt2*-knockout livers but not in brains (Fig. 1b). These observations suggest that mitochondrial respiration defects are induced in the livers but not the brains of *Shmt2*-knockout E13.5 embryos.

**Figure 2:**
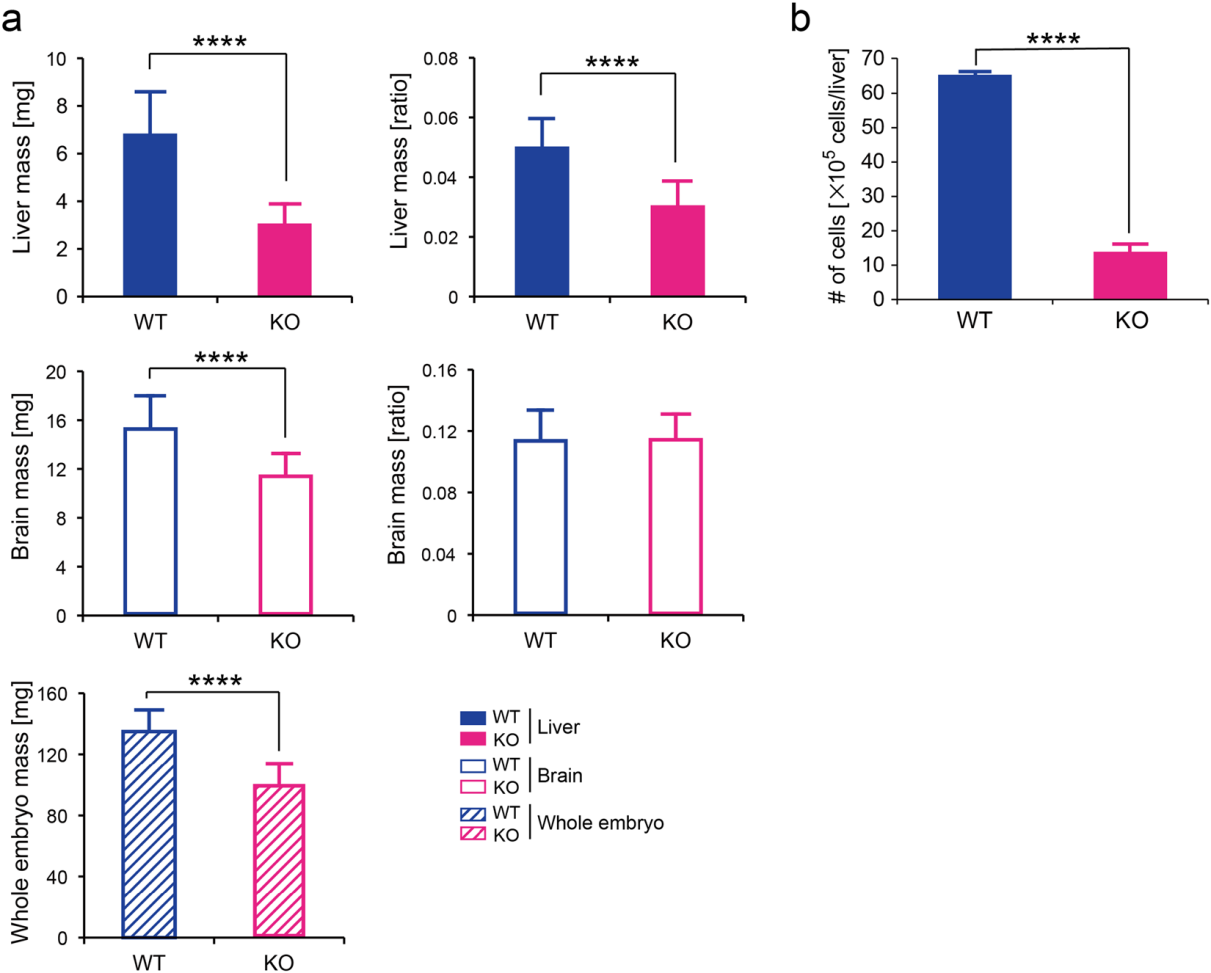
Effects of *Shmt2* knockout on the growth of livers and brains from E13.5 embryos. (**a**) Comparison of the weights of tissues or whole embryos between wild-type and *Shmt2*-knockout mice. WT, wild-type foetal livers (filled blue bars), wild-type foetal brains (open blue bars), and wild-type whole embryos (a hatched blue bar). KO, *Shmt2*-knockout foetal livers (filled red bars), *Shmt2*-knockout foetal brains (open red bars), and *Shmt2*-knockout whole embryos (a hatched red bar). Data represent the means ± S.D. ****P < 0.0001, Student’s *t* test (WT, n = 26; KO, n = 22). (**b**) Comparison of the total numbers of cells between wildtype and *Shmt2*-knockout foetal livers. All cells in each liver from an E13.5 embryo were suspended in 1 mL of PBS containing 5% FBS by a syringe with a 26-gauge needle. WT, wildtype foetal livers (filled blue bars); KO, *Shmt2*-knockout foetal livers (filled red bars). Data represent the mean ± S.D. ****P < 0.0001, 2-way ANOVA (WT, n = 3; KO, n = 4).

Next, to examine whether foetal tissues without functional *Shmt2* exhibit growth retardation, we compared the weights of wild-type and *Shmt2*-knockout E13.5 whole embryos and their tissues. Although a decrease in weight was commonly observed in *Shmt2*-knockout whole embryos and their tissues, a significant decrease in the ratio of tissue weight per body weight was observed in foetal livers but not in foetal brains (Fig. 2a). Heterozygous *Shmt2* m/+ embryos did not show decreases in the weights of tissues and whole embryos (Fig. S2), suggesting the absence of a gene-dose effect.

The induction of growth retardation in *Shmt2*-knockout foetal livers was supported by comparison of the total numbers of cells between wild-type and *Shmt2*-knockout foetal livers, because the results showed their significant decrease in *Shmt2*-knockout foetal livers (Fig. 2b). Therefore, *Shmt2* knockout possibly induces both mitochondrial respiration defects and growth retardation preferentially in foetal livers.

Given that the foetal liver is the primary site for erythropoiesis during embryogenesis^23^, growth retardation of erythroblasts in foetal livers might result in the induction of anaemia. A question that then arises is whether mitochondrial respiration defects in foetal livers might also be responsible for the induction of anaemia.

### Arrest of erythroblast differentiation in *Shmt2*-knockout foetal livers

**Figure 3:**
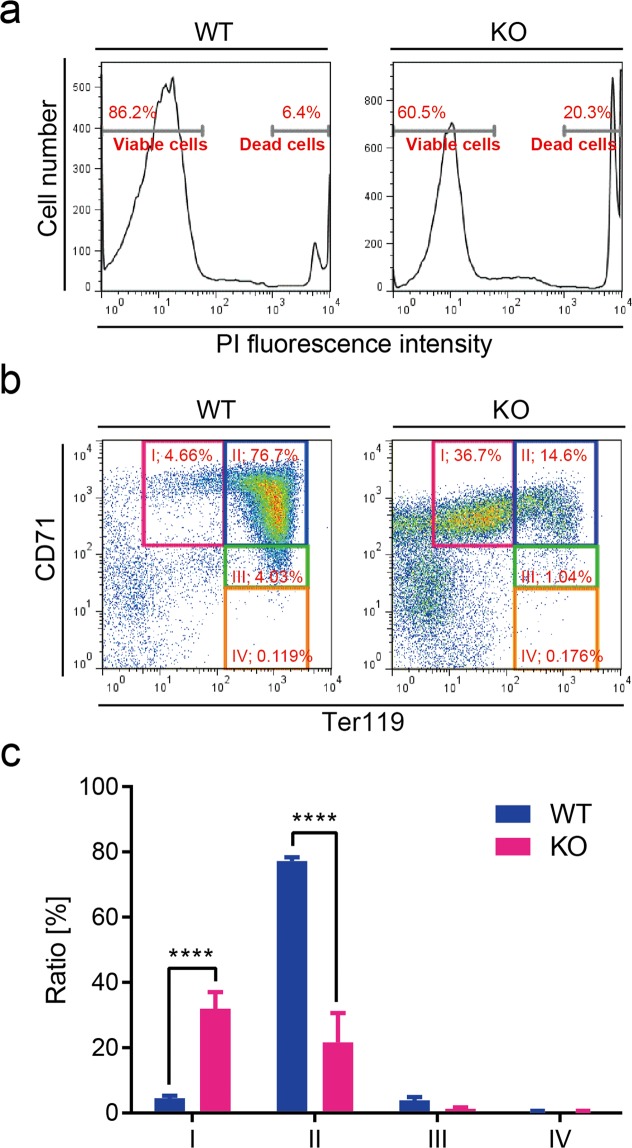
Flow cytometric analysis of liver cells from wild-type and *Shmt2*-knockout E13.5 embryos. (**a**) Dead cells from wild-type and *Shmt2*-knockout foetal livers were stained with PI and separated from viable cells using flow cytometric analysis. The same number (3 × 10^4^) of viable cells were then analysed using CD71/Ter119 flow cytometric assay. Representative results (**b**) and quantitative evaluation (**c**) of flow cytometric analysis of foetal liver cells. In the E13.5 embryonic stage, foetal livers function as erythropoietic sites, with most cells in the foetal liver representing progenitors of erythrocytes. In wild-type embryos (embryos without deficient *Shmt2*), most progenitors are at stage II (a CD71^High^/Ter119^High^ subset mostly consisting of erythroblasts). In *Shmt2*-knockout embryos, >30% of cells are at the undifferentiated stage I (a CD71^High^/Ter119^Middle^ subset mostly consisting of pro-erythroblasts), indicating the arrest of erythroblast differentiation. Cells further along in differentiation [i.e., stage III (a CD71^Middle^/Ter119^High^ subset mostly consisting of reticulocytes) and stage IV (a CD71^Low^/Ter119^High^ subset mostly consisting of matured erythrocytes)], are rare, even in wild-type embryos. WT, wild-type foetal livers (solid blue bars); KO, *Shmt2*-knockout foetal livers (solid red bars). Data represent the mean ± S.D. ****P < 0.0001, 2-way ANOVA (WT, n = 3; KO, n = 4).

Our previous study^24^ and others^25^ show that mitochondrial respiration defects caused by mtDNA mutations in adult bone marrow induce anaemia as a consequence of the impaired differentiation of erythroblasts. Therefore, we examined the possibility that mitochondrial respiration defects caused by *Shmt2* disruption also induce impaired differentiation of erythroblasts in foetal livers by flow cytometric analysis. We used phycoerythrin (PE)-conjugated and allophycocyanin (APC)-conjugated antibodies against two cell-surface markers of erythrocyte differentiation [Ter119 antigen and a transferrin receptor (CD71), respectively]. CD71 is expressed in erythroblasts during early differentiation and its expression is reduced with erythroblast maturation^26^. Ter119 antigen is expressed during terminal differentiation^27^. The cells were stained with propidium iodide (PI) to identify and exclude dead cells (Fig. 3a), and the same number (3 × 10^4^) of viable cells prepared from wild-type and *Shmt2*-knockout foetal livers were collected and analysed using CD71/Ter119 flow cytometric assays (Fig. 3b,c).

We quantified three populations of foetal liver cells, CD71^high^/Ter119^middle^ (EI), CD71^high^/Ter119^high^ (EII), and CD71^middle^/Ter119^high^ (EIII), which mainly correspond to pro-erythroblasts, erythroblasts, and reticulocytes, respectively. The results indicated that the differentiation of pro-erythroblasts to erythroblasts was significantly impaired in *Shmt2*-knockout foetal livers (Fig. 3b,c). Foetal livers did not include populations of CD71^low^/Ter119^high^ (EIV; matured erythrocytes), regardless of whether they were derived from wild-type or *Shmt2*-knockout embryos. Moreover, we observed a significant increase in the population of cells not belonging to stages EI through EIV in *Shmt2*-knockout foetal livers (Fig. 3b), partly reflecting the impaired differentiation of erythroid progenitors to pro-erythroblasts.

We were unable to estimate mitochondrial respiratory protein levels in each stage of erythropoiesis due to extremely low numbers of cells in stages EI, EIII, and EIV (Fig. 3b). However, the results in Fig. 1 reflect developing erythroblasts, because >85% of viable cells in E13.5 foetal livers represent developing erythroblasts belonging to stages EI through EIV (Fig. 3b). Given that mitochondrial respiration defects induce impaired differentiation of erythroblasts in adult bone marrow^24,25^, it is also possible that they could induce impaired differentiation of erythroblasts in *Shmt2*-knockout foetal livers (Fig. 3). Therefore, not only growth retardation (Fig. 2) but also mitochondrial respiration defects and the resulting arrest of erythroblast differentiation (Fig. 3) induced erythroblast depletion in foetal livers and were responsible for the manifestation of anaemia in *Shmt2*-knockout embryos.

Subsequent questions include how the disruption of *Shmt2* induces mitochondrial respiration defects and growth retardation and why these abnormalities occur preferentially in the livers but not the brains, even though both tissues are derived from *Shmt2*-knockout E13.5 embryos.

### Metabolomic disorders underlying anaemia in *Shmt2*-knockout embryos

**Figure 4:**
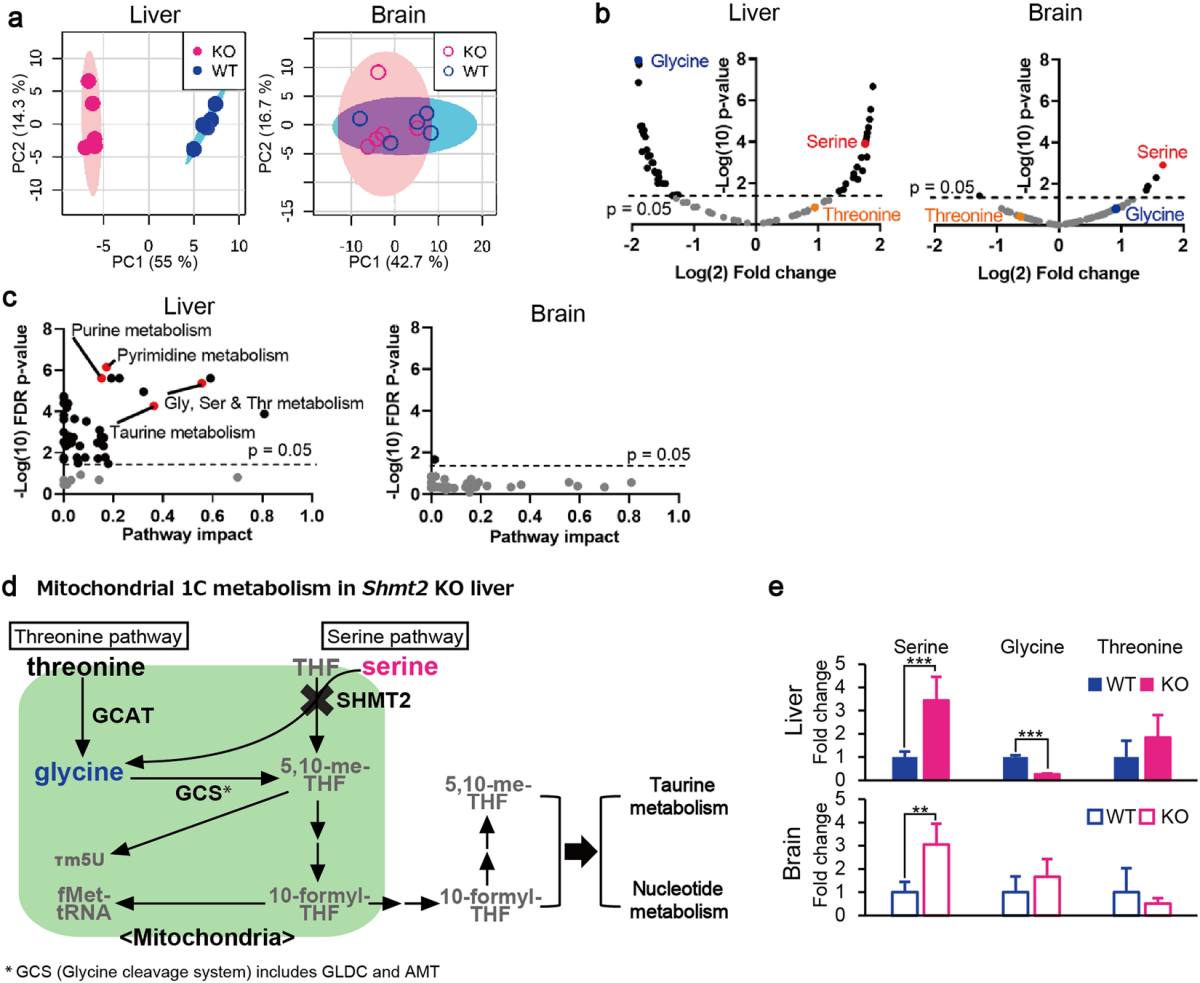
Metabolomic profiles of livers and brains from wild-type and *Shmt2*-knockout E13.5 embryos. (**a**) PCA of livers and brains to compare metabolomic data between wild-type and *Shmt2*-knockout E13.5 embryos. WT, wild-type foetal livers (closed blue circles), wild-type foetal brains (open blue circles); KO, *Shmt2*-knockout foetal livers (closed red circles), *Shmt2*-knockout foetal brains (open red circles). (**b**) Volcano plots of metabolomic changes caused by *Shmt2* knockout in livers and brains from E13.5 embryos. Broken lines, threshold of significance; red dots, serine; blue dots, glycine; orange dots, threonine; black dots, metabolites exhibiting significant changes; grey dots, metabolites without significant changes. (**c**) Pathway analysis of metabolomic changes caused by *Shmt2* knockout in livers and brains from E13.5 embryos. Broken lines, threshold of significance; black dots, pathways with significant changes; red dots, pathways involved in 1C metabolism with significant changes, including taurine metabolism, purine metabolism, pyrimidine metabolism, and glycine, serine, and threonine metabolism; and grey dots, pathways without significant changes. Pathway impact represents a combination of the centrality and pathway enrichment results. (**d**) A scheme of mitochondrial 1C metabolism in the livers of *Shmt2*-knockout E13.5 embryos. A red character denotes a metabolite that increased; a blue character denotes a metabolite that decreased; a black character denotes a metabolite that was unchanged in foetal liver without functional *Shmt2*; grey characters denote metabolites not quantified in this study. THF, tetrahydrofolate; 5,10-me-THF, 5,10-methylenetetrahydrofolate; τ m5U, 5-taurinomethyluridine; fMet, formylmethionine. (**e**) Effects of *Shmt2* knockout on concentrations of serine, glycine, and threonine in livers or brains from E13.5 embryos. WT, wildtype foetal livers (filled blue bars) and wild-type foetal brains (open blue bars); KO, *Shmt2*-knockout foetal livers (filled red bars) and *Shmt2*-knockout foetal brains (open red bars). Data represent the mean ± S.D. *P < 0.05; **P < 0.01, ***P < 0.001 (WT, n = 5; KO, n = 5).

**Figure 5:**
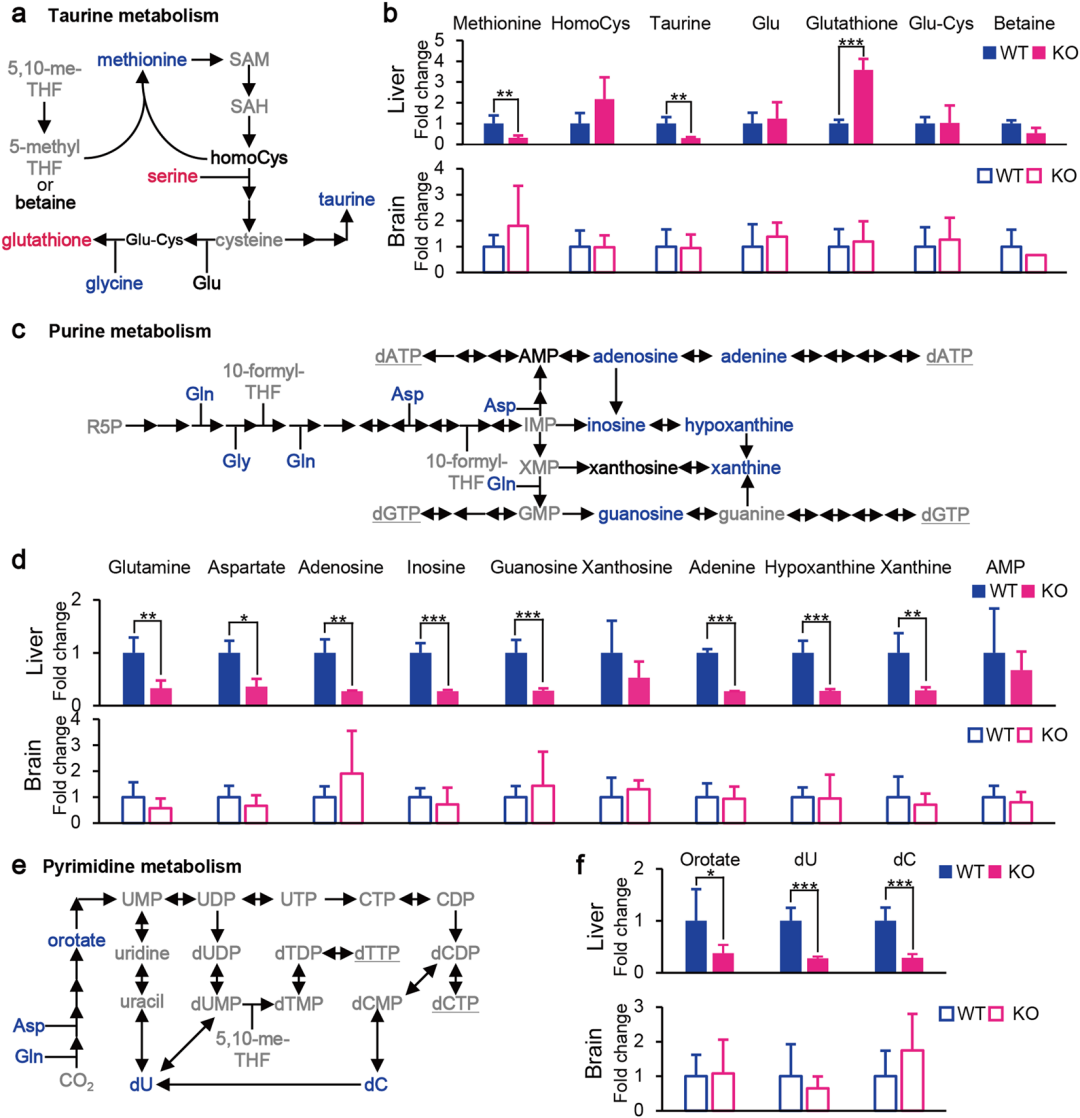
Effects of *Shmt2* knockout on taurine, purine, and pyrimidine metabolism in livers and brains from E13.5 embryos. (**a**) A scheme of taurine metabolism in livers of *Shmt2-*knockout E13.5 embryos. (**b**) Effects of *Shmt2* knockout on the concentrations of metabolites included in taurine metabolism on the livers or brains from E13.5 embryos. (**c**) A scheme of purine metabolism in livers of *Shmt2-*knockout E13.5 embryos. (**d**) Effects of *Shmt2* knockout on the concentrations of metabolites included in purine metabolism on livers or brains from E13.5 embryos. (**e**) A scheme of pyrimidine metabolism in livers of *Shmt2-*knockout E13.5 embryos. (**f**) Effects of *Shmt2* knockout on the concentrations of metabolites included in pyrimidine metabolism on livers or brains from E13.5 embryos. Red characters denote metabolites that increased; blue characters denote metabolites that decreased; black characters denote metabolites unchanged in foetal liver without functional *Shmt2*; and grey characters denote metabolites not quantified in this study. WT, wild-type foetal livers (solid blue bars) and wild-type foetal brains (open blue bars); KO, *Shmt2*-knockout foetal livers (solid red bars) and *Shmt2*-knockout foetal brains (open red bars). Data represent the mean ± S.D. *P < 0.05; **P < 0.01, ***P < 0.001 (WT, n = 5; KO, n = 5).

To answer these questions, we compared metabolomic profiles between the foetal livers of wild-type and *Shmt2*-knockout embryos and between the foetal brains of wild-type and *Shmt2*-knockout embryos and examined the tissue-specific effects of *Shmt2* disruption on metabolomic profiles via targeted metabolomics using quantitative mass spectrometry (Figs 4 and 5). Because >85% of viable cells in E13.5 foetal livers corresponded to developing erythroblasts (Fig. 3b), the metabolic profile of E13.5 foetal livers should reflect mostly the metabolic status of developing erythroblasts but not hepatic cells.

Principal component analysis (PCA) (Fig. 4a) showed clear separation of the global metabolomes of wild-type and *Shmt2*-knockout foetal livers, whereas those of wild-type and *Shmt2*-knockout foetal brains overlapped with one another. Moreover, *Shmt2* disruption significantly affected 50 metabolites (Fig. 4b) and 42 metabolic pathways (Fig. 4c) in foetal livers, whereas only five metabolites (Fig. 4b) and one pathway (Fig. 4c) were affected in foetal brains. These observations suggest that *Shmt2* is involved in substantially more pathways in foetal livers than in foetal brains. Furthermore, pathway analysis (Fig. 4c) showed that *Shmt2* disruption in foetal livers significantly affected various metabolic pathways involved in 1C metabolism, such as glycine, serine, and threonine metabolism, taurine metabolism, and nucleotide metabolism (purine and pyrimidine metabolism).

Of the pathways involved in 1C metabolism affected by *Shmt2* disruption in foetal livers, glycine, serine, and threonine metabolism (Fig. 4c) can be explained by the fact that SHMT2 catalyses the conversion of serine and tetrahydrofolate (THF) to glycine and 5,10-methylene-THF (Fig. 4d). Therefore, *Shmt2* disruption would be expected to induce elevated levels of serine and decreased levels of glycine. As expected, foetal livers without functional *Shmt2* showed increased amounts of serine and decreased amounts of glycine (Fig. 4e). By contrast, the foetal brains without functional *Shmt2* showed increased levels of serine but did not show decreased levels of glycine (Fig. 4e). These observations could be explained by postulating either a decreased utilization of glycine or the presence of additional glycine stores supplying pathways in foetal brains, such as the threonine pathway (Fig. 4d), which might rescue foetal brains from manifesting metabolomic defects by the loss of *Shmt2* (Fig. 4a).

With respect to taurine metabolism (Figs 4c,d and 5a,b) and nucleotide metabolism (Figs 4c,d and 5c–f) affected by *Shmt2* disruption in foetal livers, the loss of functional *Shmt2* might be responsible for suppressing the production of 1C units. Given that the production of active forms of THFs, such as 5,10-methenyl-THF and 10-formyl-THF, requires 1C units^11^, their deprivation by *Shmt2* disruption in foetal livers would result in deprivation of taurine and nucleotide levels (Fig. 5).

Deprivation of taurine in foetal livers without functional *Shmt2* (Fig. 5a,b) reduced the levels of subunits of mitochondrial respiratory chain complexes (Fig. 1a), because taurine is required for normal translation in the mitochondria^11^. Recent studies proposed that knockout of *SHMT2* in human cells induces mitochondrial respiration defects as a result of deprivation of taurinomethyl-uridine base^15^, and that taurine starvation causes mitochondrial respiration defects via downregulation of mitochondrial tRNA taurinomethylation^28^. Therefore, reduced levels of taurine exclusively observed in foetal livers without functional *Shmt2* (Fig. 5a,b) would subsequently decrease levels of the subunits of mitochondrial respiratory chain complexes (Fig. 1) via the insufficient taurinomethylation of mitochondrial tRNA, whereas foetal brains without functional *Shmt2* did not show these abnormalities.

Deprivation of nucleotides in foetal livers lacking functional *Shmt2* (Fig. 5c–f) would be responsible for growth retardation (Fig. 2), because nucleotides are required for cell division. We observed a decrease in the quantity of substrates and precursors for purines (Fig. 5c,d) and pyrimidines (Fig. 5e,f) exclusively in foetal livers without functional *Shmt2*. By contrast, foetal brains without functional *Shmt2* did not show these abnormalities, suggesting that tissue-specific reductions in nucleotide metabolisms might induce significant growth retardation of foetal livers lacking functional *Shmt2* (Fig. 2) as a consequence of the insufficient production of nucleotides (Fig. 5d,f) required for rapid cell division. Therefore, different foetal tissues rely differently on *Shmt2*, with the 1C metabolome of foetal livers being especially sensitive.

Given that mouse embryonic stem cells depend entirely on the threonine pathway for 1C metabolism rather than the serine pathway^29^, it is probable that most mouse embryonic tissues also use the threonine pathway (Fig. 4d) to produce taurine and nucleotides required for mitochondrial respiratory function and cell division, respectively. We examined whether foetal livers additionally activate the serine pathway (Fig. 4d) by upregulation of *Shmt2* in response to increasing demands for 1C sources to support rapid cell division and cell differentiation required for erythropoiesis in developing 13.5E foetal livers. As expected, the results showed that the levels of *Shmt2* involved in the serine pathway were significantly higher in foetal livers than foetal brains (Fig. S3a). However, disruption of *Shmt2* did not induce compensatory upregulation of *Gcat* involved in the threonine pathway or *Gldc* and *Amt* involved in the glycine cleavage system (GCS) in both foetal livers and brains (Fig. S3b), suggesting that foetal brains produce sufficient 1C sources without upregulation of these genes, even in the absence of *Shmt2*. Therefore, dependence on the serine pathway in foetal livers is much higher than that in foetal brains and represents one of the reasons why foetal livers are significantly influenced by *Shmt2* disruption.

## Discussion

This study identified potential mechanisms by which *Shmt2* disruption induces mitochondrial respiration defects and growth retardation and the manner in which these abnormalities are preferentially manifested in the livers of *Shmt2*-knockout E13.5 embryos and induce anaemia.

The livers but not the brains from *Shmt2*-knockout E13.5 embryos exhibit mitochondrial respiration defects and growth retardation. Because E13.5 foetal liver is an erythropoietic tissue^23^, growth retardation of foetal livers without functional *Shmt2* directly induces the depletion of erythroblasts and the manifestation of anaemia. Furthermore, mitochondrial respiration defects in foetal livers without functional *Shmt2* might also induce the depletion of erythroblasts by the arrest of erythroblast differentiation. This assertion is supported by our previous finding^24^ that anaemia is induced by the transplantation of adult bone marrow cells exhibiting mitochondrial respiration defects caused by the inclusion of mtDNA harbouring a large-scale deletion mutation^30,31^ into irradiated normal mice as a consequence of the differentiation arrest of erythroblasts from transplanted bone marrow cells. Elevated mutagenesis of mtDNA and the resulting mitochondrial respiration defects have also been shown to arrest erythroblast differentiation and induce anaemia in adult mice^25^.

Moreover, mitochondrial respiration defects not only impair cell differentiation but also cell division, given that growth retardation was induced in mouse tumour cells expressing the respiration defects^32,33^ by introduction of mouse mtDNA harbouring pathogenic mutations^30,34^. Therefore, while growth retardation in foetal livers induces the depletion of erythroblasts, mitochondrial respiration defects also induce the depletion of erythroblasts via inhibition of erythroblast differentiation and division, resulting in the manifestation of anaemia in *Shmt2*-knockout embryos.

Metabolomic analysis revealed the potential mechanisms by which the disruption of *Shmt2* induces mitochondrial respiration defects and growth retardation. In *Shmt2*-knockout embryos, we observed liver-specific alterations in metabolic pathways that depend on active THF-driven 1C metabolism and facilitate the synthesis of taurine and nucleotides. Specifically, we found decreased levels of taurine, which is required for translation in mitochondria and thereby for mitochondrial respiratory function. Moreover, downregulation of *de novo* nucleotide synthesis induces growth retardation^11^. Therefore, abnormalities in these 1C cycle-dependent pathways likely explain the defects observed in mitochondrial respiratory function and growth.

Metabolomic analysis also revealed the reason why mitochondrial respiration defects and growth retardation occur preferentially in the livers but not the brains of *Shmt2*-knockout E13.5 embryos (Figs 4 and S1). The serine pathway includes *Shmt2* and converts serine and THF to glycine and 5,10-me-THF to produce taurine and nucleotides required for mitochondrial respiratory function and cell division, respectively. By contrast, the threonine pathway does not include *Shmt2* and converts threonine to glycine and produces 5,10-me-THF via the GCS, resulting in the production of taurine and nucleotides (Fig. 4d). Our results suggest that foetal brains use the threonine pathway to supply glycine (Fig. 4d) and are able to escape from mitochondrial respiration defects and growth retardation by the conversion of glycine to 5,10-me-THF via the GCS, thereby eluding the manifestation of NTDs. This explanation is supported by findings that the disruption of either *Gldc*^21^ or *Amt*^19^, both of which are involved in the GCS, induces NTDs and embryonic lethality.

Therefore, mouse embryos appear to show lethality via at least two tissue-specific crises and exhibit either NTDs or anaemia by the disruption of the genes involved in mitochondrial 1C metabolism and the resulting deprivation of their products. The first crisis is the occurrence of NTDs by the disruption of *Gldc*^21^ or *Amt*^19^, and the second crisis is the occurrence of anaemia by the disruption of *Shmt2*^14^ or *Mhtfd2*^18^. Embryos without functional *Shmt2* can overcome the first crisis by exploiting the threonine pathway and the GCS, which do not involve *Shmt2*, as sources of 1C products in the neural tube.

However, *Shmt2*-knockout embryos eventually show lethality resulting from the second crisis (i.e., the manifestation of anaemia). Increasing demands for the products of 1C metabolism in foetal livers due to erythropoiesis activate the serine pathway, which involves *Shmt2*. Therefore, foetal livers without functional *Shmt2* manifest abnormalities that include anaemia. In the case of *Mthfd2*-knockout mouse embryos, their brains do not show NTDs^18^ and are capable of growing with the help of *Mthfd2L*, which corresponds to a paralogous gene of *Mthfd2* and thus compensates for disrupted *Mthfd2*. By contrast, because of increasing demands for the products of 1C metabolism required for erythropoiesis in foetal livers, *Mthfd2L* would not be sufficient to compensate for disrupted *Mthfd2* due to its lower activity relative to that of *Mthfd2*, resulting in growth retardation and the manifestation of anaemia^18^.

This study elucidated the potential mechanisms by which the disruption of *Shmt2* induces embryonic anaemia in *Shmt2*-knockout mouse embryos. *Shmt2* deficiency induced foetal-liver-specific downregulation of 1C sources required for mitochondrial respiratory function and cell division, resulting in manifestation of mitochondrial respiration defects and growth retardation, respectively. Given that livers function to produce erythroblasts in mouse E13.5 developing embryos, these abnormalities could be responsible for deprivation of erythroblasts and manifestation of embryonic anaemia. However, to provide direct evidence that *Shmt2* deficiency induces depletion of erythroblasts in foetal livers as a consequence of downregulation of 1C sources, further investigation is required to show a rescue effect in *Shmt2*-knockout embryos by supplementation of 1C sources, such as formate and glycine, to pregnant mice.

We previously proposed that age-related mitochondrial respiration defects in human fibroblasts from elderly subjects^8,10^ are the results of epigenetic controls, which induce age-associated downregulation of nuclear DNA-encoded genes, such as *SHMT2* involved in 1C metabolism^14^. The results in this study also suggest that age-associated downregulation of *SHMT2* would furthermore control age-related growth retardations, as well as mitochondrial respiration defects, in human fibroblasts from elderly subjects. Therefore, activation of *SHMT2* or uptake of certain supplementary 1C sources, such as formate and glycine, might thwart the manifestation of age-related disorders. Moreover, administration of these supplements to pregnant mothers might rescue embryonic anaemia caused by inactivation of *SHMT2*. By contrast, activation of *SHMT2* or intake of these supplements might enhance tumour growth due to the fact that *SHMT2* is activated in certain human tumour cells, and that its disruption suppresses tumour growth, as well as respiratory function^15,16^. Therefore, it appears to be controversial whether the activation of *SHMT2* or intake of these supplements extends lifespan by restoring mitochondrial respiratory function and cell division or shortens lifespan by the activation of tumour growth and tumour progression.

To resolve this controversial issue, further investigation is required to examine the lifespan of and tumour formation frequency in heterozygous *Shmt2* m/+ mice, which express half the level of SHMT2 protein but do not experience embryonic lethality and anaemia^14^.

## Methods

### Ethics statement

All animal experiments were performed in accordance with protocols approved by the Institutional Animal Care and Use Committee of University of Tsukuba, Japan (Permit Number: 15-313).

### Genotyping of mice with and without *Shmt2*

For genotyping of wild-type and *Shmt2*-knockout mice, DNA samples from the tails of adult mice or embryos were prepared using the Maxwell 16 system (Promega, Madison, WI, USA). PCR was performed using a TaKaRa Ex Taq DNA polymerase Hot Start version (Takara Bio, Shiga, Japan) and the following primer set: 5′-gag ttg acc aaa act gcc ct-3′, and 5′-tca agc ccc ata aac tgg tc-3′. The amplified samples were digested with *Xcm*I to identify a mutation in *Shmt2*^14^.

### Western blot analysis

Proteins separated by SDS-polyacrylamide gel electrophoresis in 8% or 12% gels were transferred to polyvinylidene difluoride (PVDF) membranes that were blocked with PVDF blocking reagent for Can Get Signal (Toyobo, Osaka, Japan) for 1 h then incubated with primary antibodies against SHMT2 (1:1,000; #12762; Cell Signaling Technology, Danvers, MA, USA), NDUFS4 (1:1,000; ab139178; Abcam, Cambridge, UK), NDUFA9 (1:1,000; ab14713; Abcam), MT-CO1 (1:1,000; ab14705; Abcam), SDHA (1:1,000; #11998S; Cell Signaling Technology), or β-ACTIN (1:5,000; A1978; Sigma-Aldrich, St. Louis, MO, USA) over night at 4 °C [Can Get Signal immunoreaction enhancer solution 1 (Toyobo) was used for dilution]. The membranes were then incubated with horseradish peroxidase-conjugated secondary antibodies against rabbit IgG (1:10,000; G-21234; Thermo Fisher Scientific, Waltham, MA, USA) or mouse IgG (1:10,000; G-21040; Life Technologies/Thermo Fisher Scientific, Carlsbad, CA, USA) for 1 h at room temperature [Can Get Signal immunoreaction enhancer solution 2 (Toyobo) was used for dilution]. Bands were detected with a bio-imaging analyser (EZ-Capture ST; ATTO, Tokyo, Japan) using ECL Select western blotting detection reagent (GE Healthcare, Little Chalfont, UK).

### Blue native polyacrylamide gel electrophoreses (PAGE)

The crude mitochondrial fraction was isolated from homogenates of foetal liver and brain by centrifugation (900 *g* for 5 min), followed by collection of the supernatant and re-centrifugation at 5,000 *g* for 10 min. Isolated mitochondrial fractions were solubilized with solubilisation buffer [1.5 M aminocaproic acid and 50 mM Bis-Tris (pH 7.0) containing 1.5% n-dodecyl-β-D-maltoside] and centrifuged at 40,000 *g* for 30 min. The resulting supernatants were subjected to electrophoresis (8 μg/lane) conducted using NativePAGE 3–12% Bis-Tris protein gels (Thermo Fisher Scientific, Waltham, MA). To detect respiratory chain complexes, gels were incubated in staining buffer [2.5 mg/mL Coomassie Brilliant Blue R250 (CBBR) in 45% MeOH/10% acetic acid] overnight. The gels were then washed with wash buffer (45% MeOH/10% acetic acid) several times until the complex bands became visible. To evaluate in-gel complex I activity, gels were incubated in assay buffer [2 mM Tris-HCl, 0.1 mg/mL NADH, and 2.5 mg/mL nitro blue tetrazolium chloride (pH 7.4)].

### Cell count and flow cytometry

Foetal liver was dispersed using a syringe with a 26-gauge needle to obtain foetal liver cells, which were suspended in 1 mL of phosphate-buffered saline (PBS) containing 5% foetal bovine serum (FBS). A part of the suspension (20 μL) was used for the cell count, and the remaining was passed through a nylon mesh (70 μm) for immunostaining with PE-conjugated anti-Ter119 (Miltenyi Biotec, Bergisch Gladbach, Germany) and APC-conjugated anti-CD71 (Thermo Fisher Scientific) antibodies for 30 min at 4 °C. After washing the cells with PBS containing 5% FBS, dead cells were stained with PI (2 μg/mL) for 10 min at 4 °C. Flow cytometric analysis using ~3 × 10^4^ viable cells from a foetal liver was performed using a FACSCalibur flow cytometer (BD Biosciences, Franklin Lakes, NJ, USA) with CellQuest software (BD Biosciences). Data were analysed with FlowJo 7.6 software (Tree Star, Ashland, OR, USA).

### Quantitative mass spectrometry based targeted metabolomics

Metabolites were extracted from ~15 mg of frozen tissues and separated using an Acquity ultra-pressure liquid chromatography system and analysed using a Xevo TQ-S triple quadrupole mass spectrometer (Waters Corporation, Milford, MA, USA). Complete descriptions of the methods, including instrument parameters and validation, are provided elsewhere^35^. The resulting targeted metabolomics data were analysed using MetaboAnalyst 3.0 (www.metabolanalyst.ca)^36,37^, log-transformed, and auto-scaled prior to statistical analysis. Separation among groups was tested by unsupervised multivariate analysis and PCA. To identify which metabolic pathways were significant with “hub” nodes, we performed metabolic pathway topological analysis. Quantitative data was mapped, and a mouse metabolite-pathway library with 82 entries was selected. The other parameters chosen were for global testing and betweenness-centrality node measurement. Pathway analysis results were displayed as a metabolome view graph.

### Quantification of mRNA levels by real-time quantitative PCR

Total RNA was extracted from the livers and brains of wild-type or *shmt2*-knockout mice using the RNeasy mini kit (Qiagen). RNA samples were reverse-transcribed using a high-capacity cDNA reverse transcription kit (Thermo Fisher Scientific). Real-time PCR was performed with GeneAce SYBR qPCR mix α (Nippon Gene, Tokyo, Japan), on an ABI PRISM 7900HT sequence detection system (Applied Biosystems, Foster City, CA, USA) and using primers for *Shmt2* (forward, ctgcagagggagaaggacag; reverse, ctcggctgcagaagttctct), *Gcat* (forward, gcctatttgaggctttgctg; reverse, gtggcggtaacggtacttgt), *Gldc* (forward, actgtggatcttcggctgtg; reverse, gaacgggctggttctcttga), *Amt* (forward, tactacacctgtggagggca; reverse, cacatatcagccccacacgt) and *Tbp* (forward, ctggaattgtaccgcagctt; reverse, atgatgactgcagcaaatcg). The relative expression levels of each mRNA were calculated using the ΔΔCt method, with *Tbp* used as an internal control.

### Statistical analysis

Data were analysed by two-sided Student’s *t* test or analysis of variance (ANOVA) or Tukey-Kramer method. A *P* < 0.05 was considered significant.

## Supplementary information

Supplementary figure
