# Supplementary material for: Disruption of the mouse Shmt2 gene confers embryonic anaemia via foetal liver-specific metabolomic disorders
Source: Sci Rep. 2019 Nov 5;9:16054. doi: 10.1038/s41598-019-52372-6 (PMC6831688; doi:10.1038/s41598-019-52372-6)
Supplement: Supplementary file 1 — Supplementary figure [file 41598_2019_52372_MOESM1_ESM.pdf]

Disruption of the mouse *Shmt2* gene confers embryonic anaemia via foetal liver-specific metabolomic disorders

Haruna Tani[1,2,†], Takayuki Mito[3,†], Vidya Velagapudi[4], Kaori Ishikawa[1,5], Moe Umehara[1], Kazuto Nakada[1,5], Anu Suomalainen[3,6,7], Jun-Ichi Hayashi[8,*]

[1] Graduate School of Life and Environmental Sciences, University of Tsukuba, 1-1-1 Tennodai, Tsukuba, Ibaraki 305-8572, Japan.
[2] Japan Society for the Promotion of Science (JSPS), 8 Ichiban-cho, Chiyoda-ku, Tokyo 102-8472, Japan
[3] Research Programs Unit, Molecular Neurology, University of Helsinki, 00290 Helsinki, Finland
[4] Metabolomics Unit, Institute for Molecular Medicine Finland FIMM, HiLIFE, University of Helsinki, 00290 Helsinki, Finland
[5] Faculty of Life and Environmental Sciences, University of Tsukuba, 1-1-1 Tennodai, Tsukuba, Ibaraki 305-8572, Japan.
[6] Department of Neurology, Helsinki University Hospital and Clinical Neurosciences, University of Helsinki, 00290 Helsinki, Finland
[7] Neuroscience Center, HiLife, University of Helsinki, 00290 Helsinki, Finland
[8] Life Science Center for Survival Dynamics, Tsukuba Advanced Research Alliance (TARA), University of Tsukuba, 1-1-1 Tennodai, Tsukuba, Ibaraki 305-8577, Japan.
[†]These authors contributed equally to this work.
[*]Correspondence and requests for materials should be addressed J.-I. H. (Tel: +81 29 853 6650; Fax: +81 29 853 6614; E-mail: jih45@biol.tsukuba.ac.jp)

Figure S1

SHMT2 Antibody

M : Maker

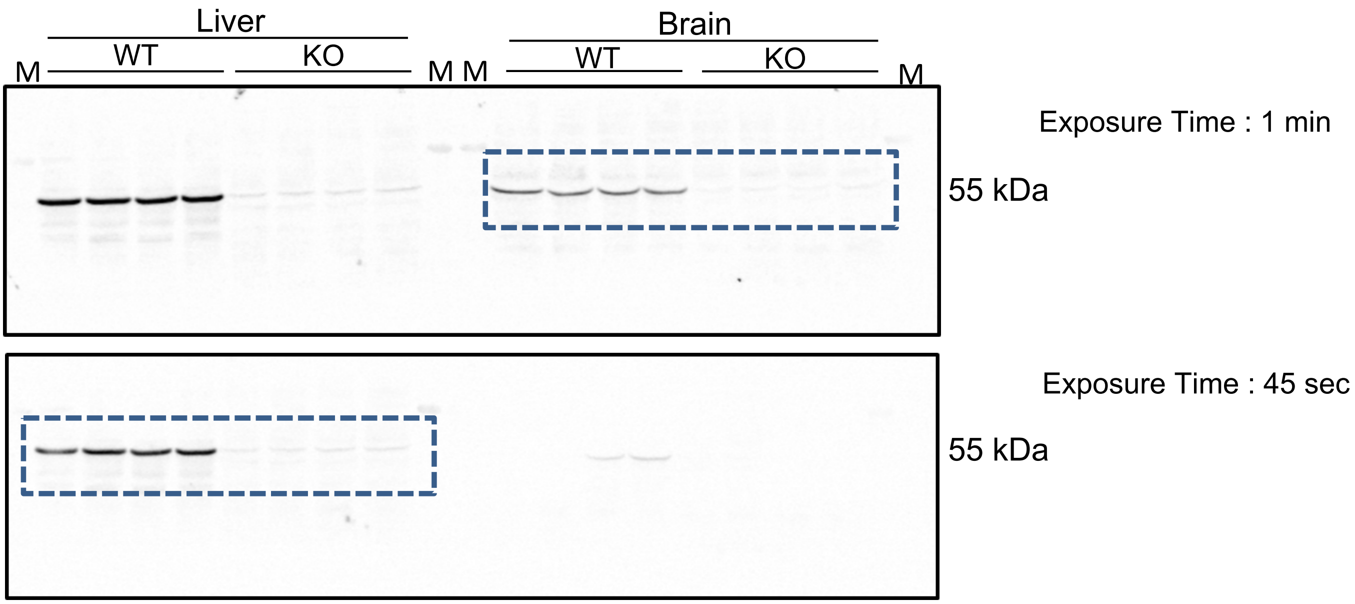

NDUFS4 Antibody

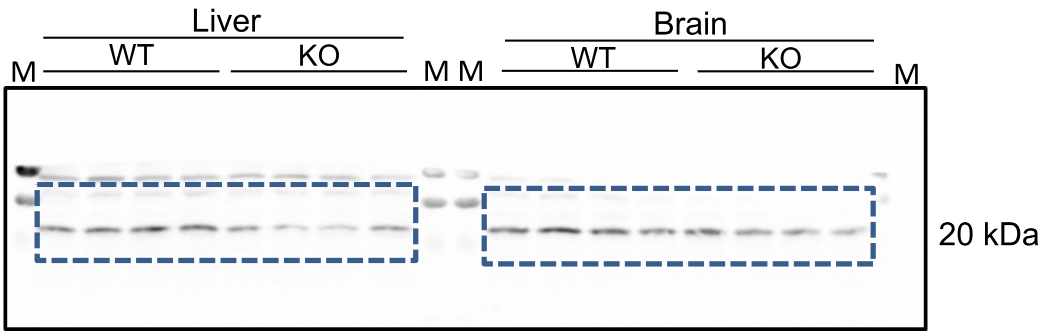

NDUFA9 Antibody

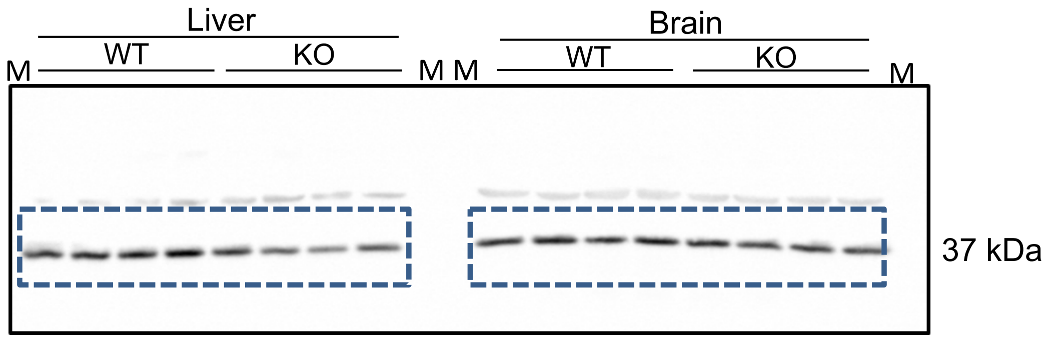

MT-CO1 Antibody

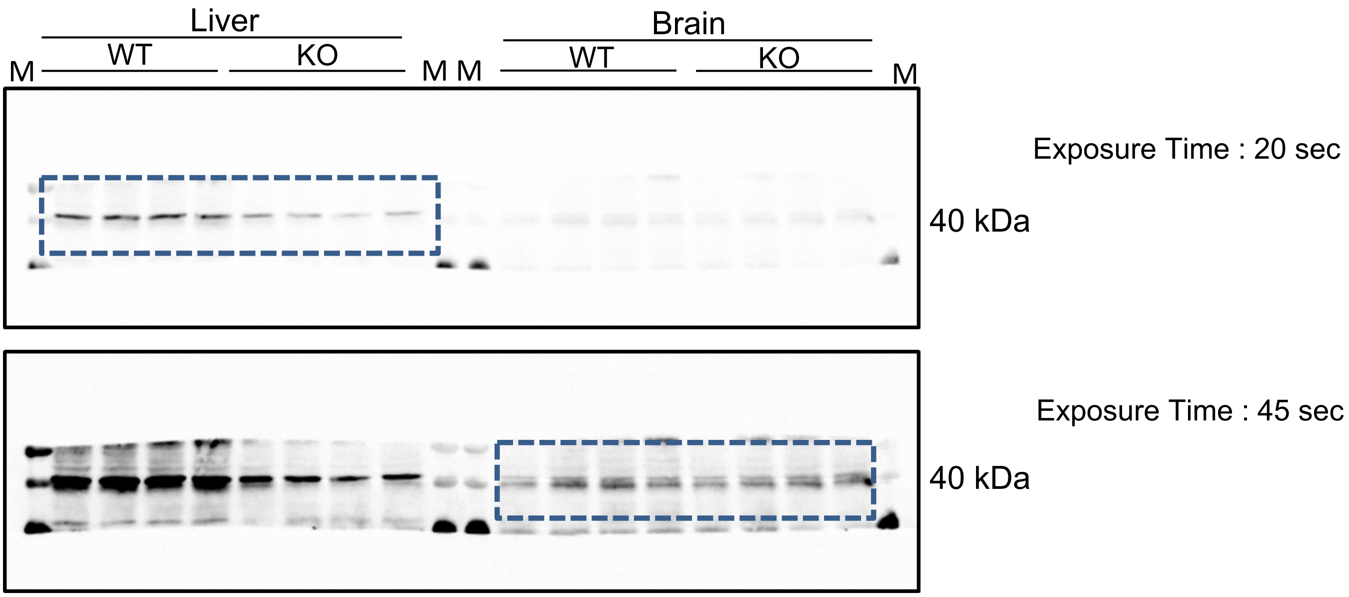

SDHA Antibody

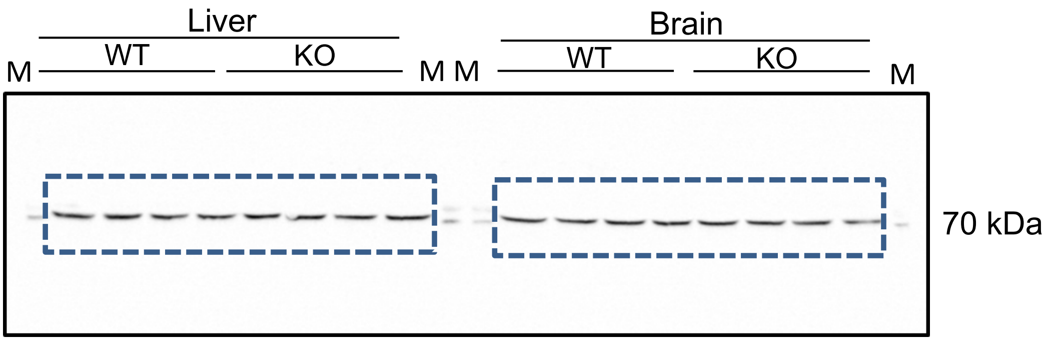

β-ACTIN Antibody

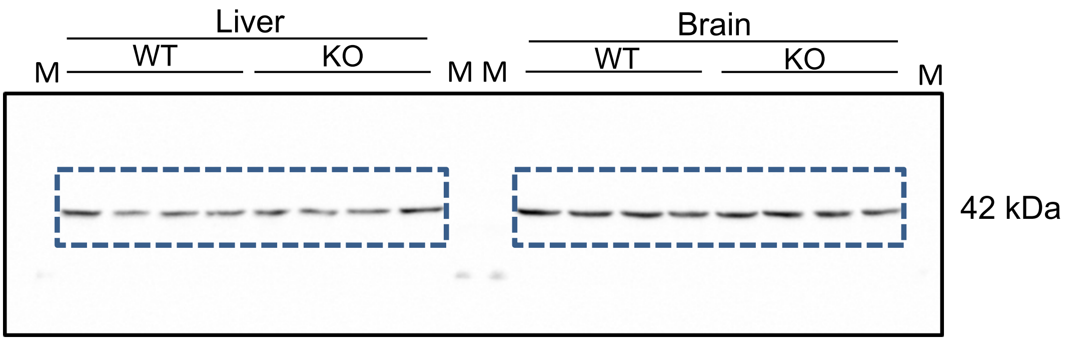

Figure S1 Full-length blots of Figure 1
Membranes were cut to enable reacting of multiple antibodies. some membranes were detected at several exposure times. The dotted lines indicate the sites used in Figure 1.

Figure S2

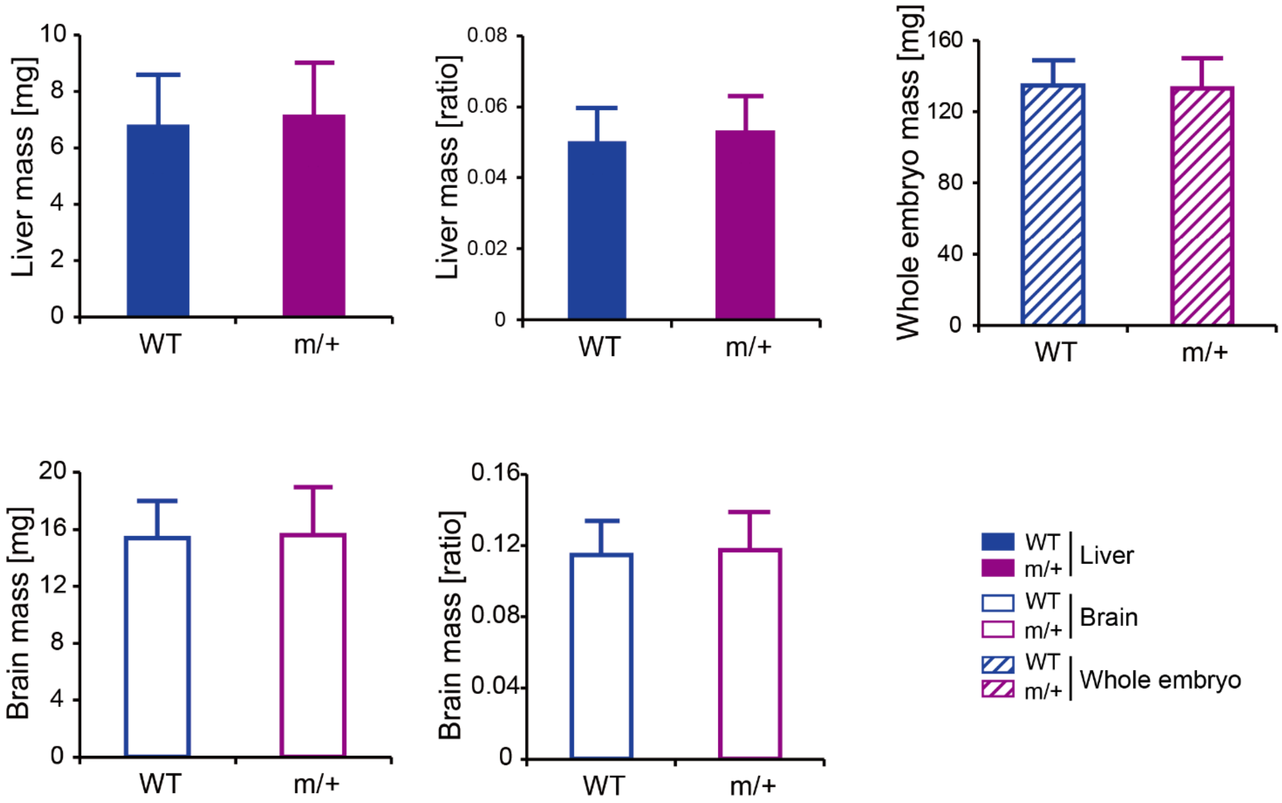

Figure S2 Effects of *Shmt2* disruption on the growth of livers and brains from E13.5 heterozygous *Shmt2* m/+ embryos. Comparison of the weights of tissues or whole embryos between wild-type and heterozygous *Shmt2* m/+ mice. WT, wild-type foetal livers (filled blue bars), wild-type foetal brains (open blue bars), and wild-type whole embryos (a hatched blue bar). m/+, heterozygous foetal livers (filled purple bars), heterozygous foetal brains (open purple bars), and heterozygous whole embryos (a hatched purple bar). Data represent the means ± S.D, Student's *t* test (WT, n=26; m/+, n=27).

Figure S3

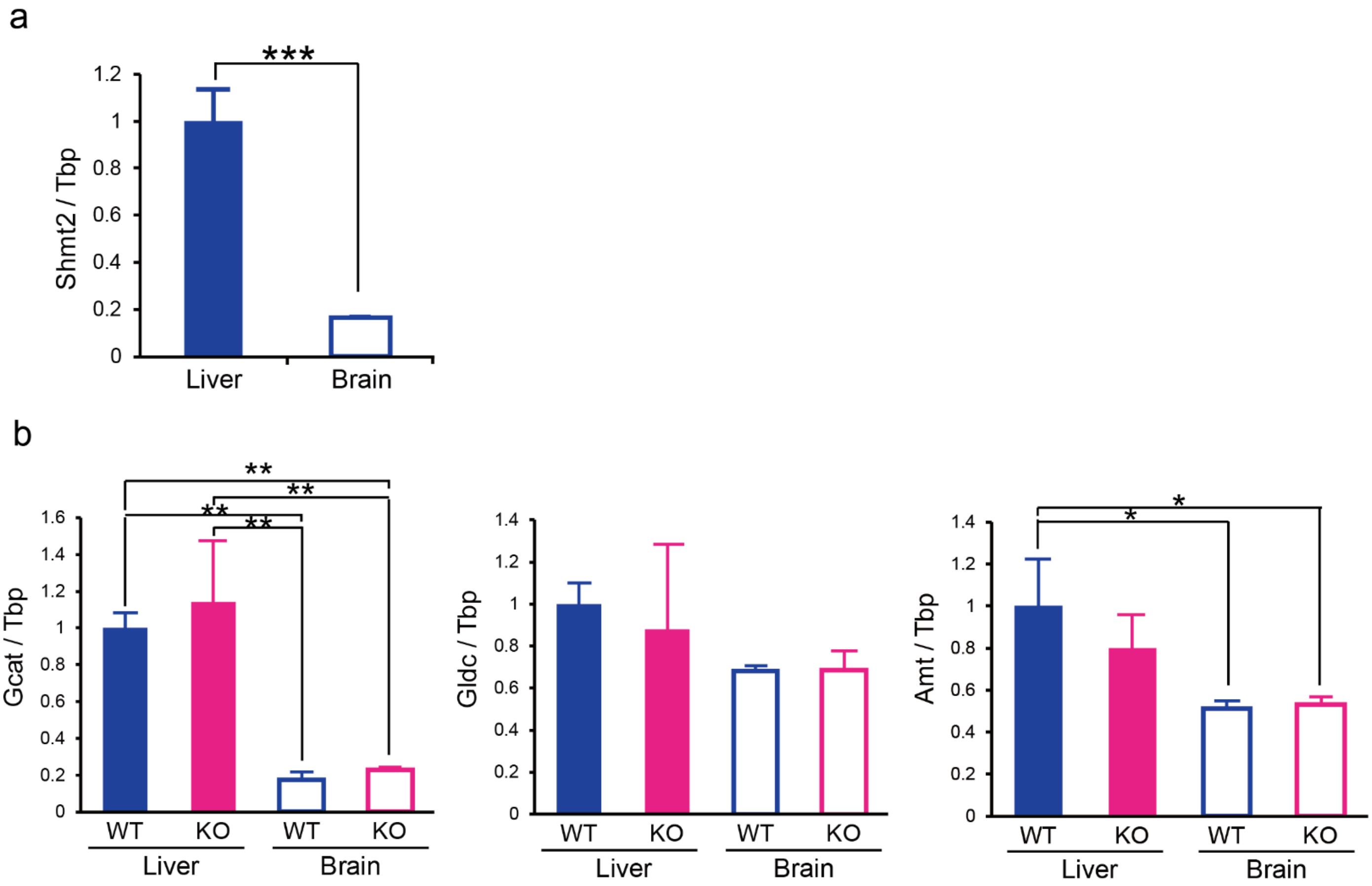

Figure S3 Real-time quantitative PCR to compare mRNA levels of genes involved in 1C metabolism. (**a**) Comparison of mRNA levels of *Shmt2* involved in the serine pathway between wildtype foetal livers and brains. (**b**) Comparison of mRNA levels of *Gcat* involved in the threonine pathway and those of *Gldc* and *Amt* involved in the GCS in wild-type and *Shmt2*-knockout foetal livers and brains. Filled blue bars, wild-type foetal livers; filled red bars, *Shmt2*-knockout foetal livers; open blue bars, wild-type foetal brains; open red bars, *Shmt2*-knockout foetal brains. Data represent the means ± S.D. *$P < 0.05$; **$P < 0.01$, ***$P < 0.001$, Tukey-Kramer method (WT, n = 3; KO, n = 3).
